# Functional characterization of the *idtF* and *idtP* genes in the *Claviceps paspali* indole diterpene biosynthetic gene cluster

**DOI:** 10.1007/s12223-020-00777-6

**Published:** 2020-02-19

**Authors:** László Kozák, Zoltán Szilágyi, László Tóth, István Pócsi, István Molnár

**Affiliations:** 1 Department of Molecular Biotechnology and Microbiology, Institute of Biotechnology, Faculty of Science and Technology, University of Debrecen, Debrecen, Hungary; 2 Teva Pharmaceutical Works Ltd., Debrecen, Hungary; 3 Southwest Center for Natural Products Research, School of Natural Resources and the Environment, University of Arizona, Tucson, USA

## Abstract

*Claviceps paspali* is used in the pharmaceutical industry for the production of ergot alkaloids. This fungus also biosynthesizes paspalitrems, indole diterpene (IDT) mycotoxins that cause significant economic losses in agriculture and represent safety concerns for ergot alkaloid manufacture. Here, we use *Agrobacterium-*mediated transformation to replace the *idtP* and the *idtF* genes in the IDT biosynthetic gene cluster of *C. paspali* with a selectable marker gene. We show that the *ΔidtP* knockout mutant produces paspaline, the first IDT intermediate of the pathway. The *ΔidtF* strain produces unprenylated IDTs such as paspalinine and paspaline. These experiments validate the function of *idtP* as the gene encoding the cytochrome P450 monooxygenase that oxidizes and demethylates paspaline to produce 13-desoxypaxilline, and that of *idtF* as the gene that encodes the α-prenyltransferase that prenylates paspalinine at the C20 or the C21 positions to yield paspalitrems A and C, respectively. In addition, we also show that axenic cultures of the wild type, the *ΔidtP* and the *ΔidtF* mutant *C. paspali* strains fail to produce an assembly of IDTs that are present in *C. paspali*–*Paspalum* spp. associations.

**Electronic supplementary material:**

The online version of this article (10.1007/s12223-020-00777-6) contains supplementary material, which is available to authorized users.

## Introduction

*Claviceps paspali* is a hypocrealean fungus that has been used in the pharmaceutical industry for decades to produce ergot alkaloids. These alkaloids serve as precursors for the manufacture of drugs that treat Parkinson’s disease and migraine (Arcamone et al. 1960; Tudzynski et al. 2001). In its natural environment, *C. paspali* forms an association with dallis grasses (*Paspalum* spp.) and produces not only ergot alkaloids but also indole diterpenes (IDTs) such as paspalitrems (Cole et al. 1977; Uhlig et al. 2014). Ingestion of grasses and grains contaminated with paspalitrem IDTs causes an array of symptoms in large animals often referred to as “Paspalum stagger,” characterized by tremor, ataxia, and convulsions. Although Paspalum stagger is rarely lethal for the intoxicated livestock, reduced body mass gain and culling due to accidents suffered by the animals as a result of uncoordinated movement lead to large losses in agriculture (Cole et al. 1977; Moyano et al. 2010; Cawdell-Smith et al. 2010). Importantly for the pharmaceutical industry, *C. paspali* produces IDTs not only when it forms associations with host plants but also in axenic cultures. The presence of IDTs complicates the isolation and purification of ergot alkaloids during downstream processing in industrial fermentations. Thus, safety concerns and process economics both led to a demand in the pharmaceutical industries for paspalitrem non-producing mutant *C. paspali* strains (Kozák et al. 2018).

**Fig. 1:**
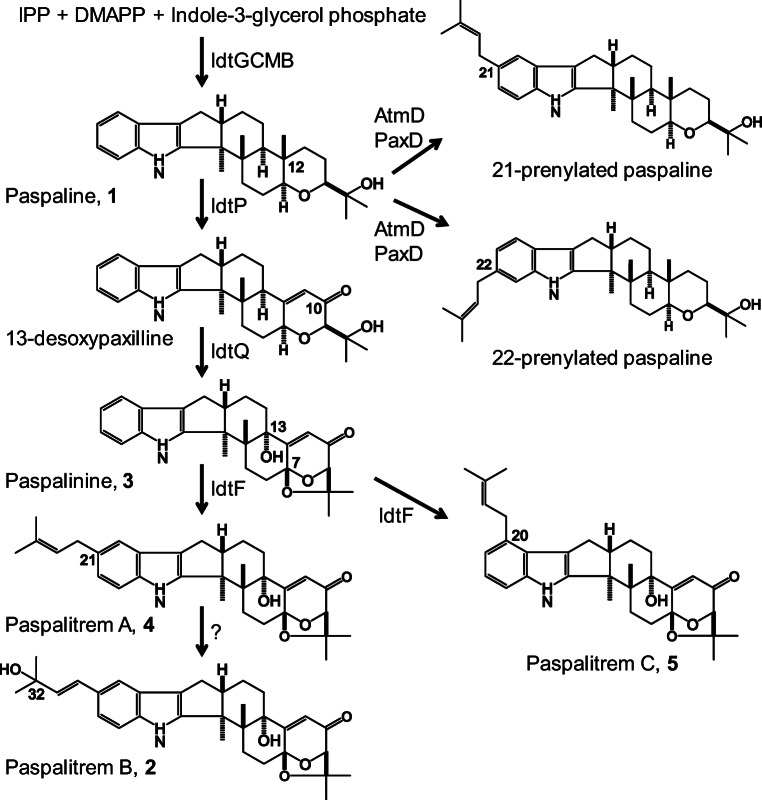
Proposed biosynthesis of paspalitrem B in *C. paspali*. Structures of reverse prenylated paspaline derivatives obtained in vitro using purified AtmD of *A. flavus* or PaxD of *P. paxilli* are also shown

Paspalitrems are derived from paspaline (**1**, Fig. 1), the simplest cyclic IDT that contains a tetracyclic diterpene moiety fused with an indole group (Kozák et al. 2019). The cyclic diterpene of paspaline is derived from geranylgeranyl diphosphate, while the indole originates from tryptophan via indole-3-glycerol phosphate (Liu et al. 2015). Tailoring of the common paspaline core by various enzymes (e.g., P450 monooxygenases, prenyltransferases, and FAD-dependent monooxygenases) yields the considerable chemical diversity within the IDT group of fungal secondary metabolites (Kozák et al. 2019).

Biosynthetic gene clusters for most of the known paspaline-derived IDTs have been identified, and the individual biochemical steps leading to the main IDT groups are well characterized (Young et al. 2005; Young et al. 2006; Nicholson et al. 2009; Tarui et al. 2014; Nicholson et al. 2015). However, IDT biosynthesis in the industrially important IDT producer *C. paspali* remained little studied. Previously, we provided functional proof for the involvement of a putative IDT biosynthetic locus of *C. paspali* DSM833, identified by genome sequencing, in the production of paspalitrems. Deletion of the *idtCBGF* genes of the paspalitrem cluster of *C. paspali* resulted in the complete abrogation of all IDT-related metabolites, while ergot alkaloid production continued undisturbed in the mutant. We also proposed a biosynthetic scheme for paspalitrem B (**2**, Fig. 1) in this fungus using bioinformatic analysis of the biosynthetic genes and by detecting IDT intermediates in fermentation extracts of the wild-type strain (Kozák et al. 2018).

During paxilline biosynthesis in *Penicillium paxilli*, the cytochrome P450 monooxygenase (PaxP) catalyzes the oxidative elimination of the pendant methyl group at C12 of the common intermediate paspaline (**1**, Fig. 1) and generates the C10 ketone to yield 13-desoxypaxilline (McMillan et al. 2003). In *P. paxilli*, deletion of *paxP* results in the accumulation of paspaline (Nicholson et al. 2015). In contrast, deletion of *janP* (encoding the corresponding PaxP orthologue) during the biosynthesis of shearinines in *P. janthinellum* results in the elimination of the production of all IDTs, including paspaline (Nicholson et al. 2015). Finally, although the PaxP orthologue TerP of *Tolypocladium album* is able to convert paspaline to 13-desoxypaxilline, during terpendole biosynthesis 13-desoxypaxilline is only a shunt metabolite, with paspaline serving as the substrate for TerQ which hydroxylates the C11 carbon, giving rise to terpendole E (Motoyama et al. 2012). The biosynthesis of lolitrems follows a similar logic in *Neotyphodium lolii/Epichloë festucaë* (Saikia et al. 2012). In *C. paspali*, the candidate enzyme catalyzing the conversion of paspaline to 13-desoxypaxilline is IdtP that shows 41% amino acid sequence identity to PaxP. Considering the above precedents, we were interested to establish whether deletion of *idtP* in *C. paspali* would lead to abrogation of IDT biosynthesis; accumulation of paspaline; or perhaps the biosynthesis of shunt paspaline derivatives modified by enzymes with broad specificity, such as a prenyltransferase that may accept paspaline for prenylation (Liu et al. 2013).

During paspalitrem biosynthesis in *C. paspali*, 13-desoxypaxilline is proposed to be converted to paspalinine (**3**, Fig. 1) by the IdtQ cytochrome P450 monooxygenase via oxidations at the C13 and C7 positions. Paspalitrems A and C (**4** and **5**, Fig. 1) are formed by the prenylation of the C21 or C20 positions of paspalinine (**3**), respectively, by a monoprenyl transferase, suggested to be IdtF (Kozák et al. 2018). In *A. flavus*, prenylation of paspalinine is catalyzed by the AtmD prenyltransferase to yield aflatrem or β-aflatrem, the structural isomers of paspalitrems A and C, respectively. Surprisingly, IdtF shows only very low similarity to AtmD (21.2% identity over 74% coverage at the amino acid level). In fact, AtmD shows higher similarity to the DmaW prenyltransferase of the ergot alkaloid biosynthetic gene cluster of *C. paspali* (27.4% identity over 92% coverage at the amino acid level). Indeed, crosstalk between different fungal biosynthetic gene clusters and localization of some secondary metabolite biosynthetic genes outside of the main cluster is often encountered. Thus, considering that IdtF is not an AtmD orthologue, experimental verification of its deduced role in paspalitrem biosynthesis in *C. paspali* is necessary.

In the current work, we set out to provide proof for the function of IdtP and IdtF by knocking out the *idtP* and *idtF* genes of *C. paspali* and comparing the IDT profiles of the mutant strains to that of the wild type*.* At the same time, we hoped that we may be able to block IDT biosynthesis at the paspaline or paspalinine stage, thus opening the way for the establishment of fermentation technologies for the large-scale production of the IDT nucleus for subsequent chemical or biosynthetic derivatization and structure—activity relationship studies for pharmaceutical drug discovery.

## Materials and methods

*C. paspali* DSM833 was used throughout this work. The maintenance of the fungus, fermentation conditions, sample preparation for IDT analysis, and genomic DNA isolation was carried out as describer earlier (Kozák et al. 2018). PCR reactions were carried out in 50 μL total volumes, containing 20 ng genomic DNA, or 1 ng plasmid DNA as the template, respectively; 0.2 mmol/L of each dNTP; 1 pmol/L of each primer; 1 μL Phusion® High-Fidelity DNA Polymerase; and 10 μL HF buffer (New England Biolabs, Ipswich, MA). Reaction conditions were as follows: 98 °C for 180 s for the initial denaturation, followed by 31 cycles of amplification (98 °C for 10 s, 55 °C for 15 s, 72 °C for 30 s/kbp), and a final extension step of 60 s/kbp at 72 °C.

**Fig. 2:**
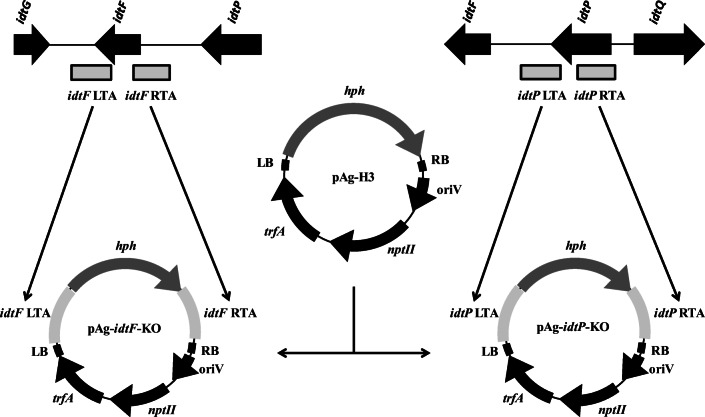
Construction of the pAg-*idtF*-KO and pAg-*idtP*-KO vectors. The left and right targeting arms (LTA and RTA, respectively) for the *idtF* and *idtP* genes, respectively, were PCR amplified from the genomic DNA of the *C. paspali* DSM833 strain. The backbone of the pAg-H3 plasmid (without the *hph* gene) and the *hph* gene alone were amplified separately and fused with the appropriate LTA and RTA amplicons using Gibson assembly. Gene symbols: *hph*, hygromycin phosphotransferase; *nptII*: neomycin phosphotransferase II (kanamycin resistance); *trfA*, plasmid replication initiation gene; oriV, vegetative origin of replication; LB and RB, left and right T-DNA border, respectively

Constructions of the pAg-*idtF*-KO and pAg-*idtP*-KO vectors for the disruption of the *idtF* and *idtP* genes, respectively, were carried out from overlapping PCR fragments using the Gibson Assembly Master Mix (New England Biolabs, Ipswich, MA) (Fig. 2). The hygromycin phosphotransferase gene (*hph*) (Gritz and Davies 1983) of the pAg-H3 vector (Zhang et al. 2003) and the rest of the vector were amplified by PCR in two separate reactions (primers are listed in Supplementary Table S1 of the Supplementary Information). The left and right targeting arms for the *idtF* and the *idtP* genes were also amplified by PCR using *C. paspali* genomic DNA as the template and appropriate primers (SI Supplementary Table S1). The four PCR amplicons (the *hph* gene, the rest of the pAg-H3 vector, and the appropriate left and right targeting sequences) were fused using the Gibson Assembly Master Mix, utilizing overlapping sequences between the adjacent DNA fragments at the 5′ ends of the primers. The resulting Gibson reaction products were transformed into *E. coli* XL1-Blue chemical competent cells (New England Biolabs, Ipswich, MA), and the transformed cells were grown on LB agar plates supplemented with 25 μg/mL kanamycin. Plasmids from kanamycin-resistant colonies were isolated using the EZ-10 Spin Column Plasmid DNA Minipreps Kit (Bio Basic Inc., Toronto, Canada) and verified by diagnostic PCR and DNA sequencing. The correctly assembled pAg-*idtF*-KO and pAg-*idtP*-KO plasmids were separately transformed into *A. tumefaciens* LBA4404 electrocompetent cells (Takara Bio Inc., Kusatsu, Japan), and the transformants were selected on LB agar plates supplemented with kanamycin (25 μg/mL) and streptomycin (50 μg/mL). *A. tumefaciens*-mediated transformation of *C. paspali*, and the selection and homogenotization of initial transformants were carried out as described previously (Kozák et al. 2018). The genotypes of the *C. paspali* transformants were validated for the expected gene knockout alleles using diagnostic PCR reactions as described in the “Results” section, using appropriate primers (SI Supplementary Table S1).

Three validated isolates were collected for each knockout mutant strain, and these were evaluated in shake flask fermentations for IDT congener production. Two isolates of each mutant strain and the wild-type strain were subsequently analyzed in detail by determining their IDT profiles in the fermentation extracts, using liquid chromatography–high resolution tandem mass spectrometry (LC-HRMS^n^) analysis using an Agilent (Santa Clara, CA) 6550 iFunnel Q-TOF mass spectrometer connected to a 1290 Infinity LC System. Experimental conditions for the fermentation, HPLC separation, and mass spectrometry were described earlier (Kozák et al. 2018). In the current work, the target compounds (paspaline **1**, paxilline, paspalinine **3**, paspalitrem A **4**, paspalitrem C **5**, and paspalitrem B **2**) were identified by comparison of their retention times and exact masses to our previous data (Kozák et al. 2018). The presence of additional paspalitrem-related IDTs (SI Supplementary Table S2) has been investigated using extracted ion chromatograms (EICs) with narrow mass windows (0.02 Da) for the analytes, with specificity ensured by the acquired high resolution MS data. The fragmentation profiles of the main IDTs isolated from *C. paspali* were described in our previous work (Kozák et al. 2018). Data shown are representative of at least two fermentation experiments with two independent isolates each per strain, in two technical replicates.

## Results

**Fig. 3:**
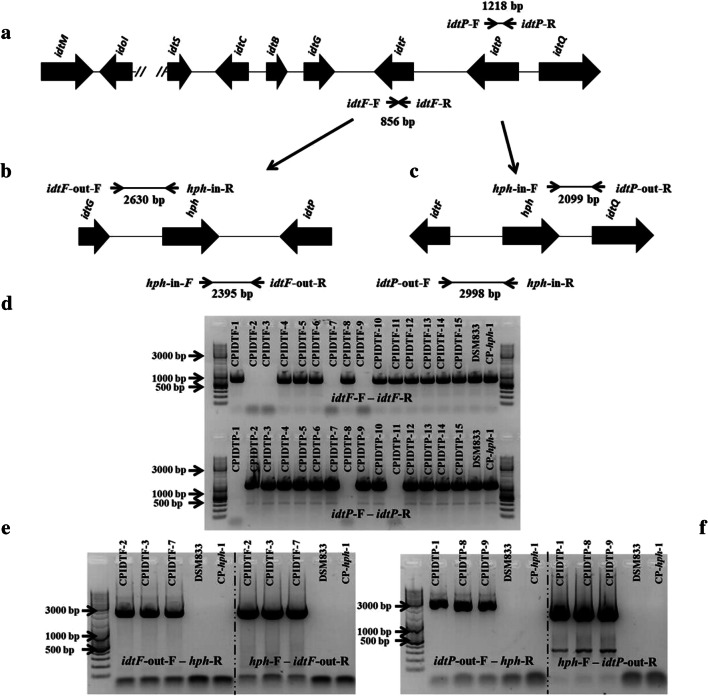
PCR validation of *C. paspali* transformants. **a** The IDT biosynthetic loci of *C. paspali* DSM833. Arrow pairs indicate the locations and the sizes (in base pairs) of the PCR amplicons that were designed to detect the intact copies of the *idtF* (primer pair: *idtF*-F–*idtF*-R) and the *idtP* genes (primer pair: *idtP*-F–*idtP*-R), respectively. **b** and **c** The *∆idtF* (**b**) or the *∆idtP* (**c**) mutant alleles, respectively, whereby the *hph* gene replaces the target gene. Arrow pairs indicate the locations and sizes (in base pairs) of the amplicons that were designed to validate the replacement. Primer pairs for the *∆idtF* allele: *idtF*-out-F–*hph*-in-R; and *hph*-in-F–*idtF*-out-R. Primer pairs for the *∆idtP* allele: *idtP*-out-F–*hph*-in-R and *hph*-in-F–*idtP*-out-R. **d** PCR analysis to detect the presence of the intact *idtF* (top row) or *idtP* gene (bottom row). DNA from the wild-type *C. paspali* DSM833 and a strain transformed with the pAg-H3 plasmid (CP-*hph*-1) are included as positive controls (intact *idtF* or *idtP* alleles). Transformants CPIDTF2, CPIDTF3, CPIDTF7, and CPIDTF9 lack the *idtF* gene. Transformants CPIDTP1, CPIDTP8, and CPIDTP11 miss the *idtP* gene. **e** and **f** PCR analyses to detect the Δ*idtF* (**e**) or the Δ*idtP* (**f**) mutant alleles, respectively. DNA from the wild-type *C. paspali* DSM833 and a strain transformed with the pAg-H3 plasmid (CP-*hph*-1) are included as negative controls (intact *idtF* or *idtP* alleles). In transformants CPIDTF2, CPIDTF3, and CPIDTF7, the *idtF* gene is replaced by the *hph* gene. In transformants CPIDTP1, CPIDTP8, and CPIDTP9, the *idtP* gene is replaced by the *hph* gene

To verify the proposed functions of the *idtP* and *idtF* genes in paspalitrem biosynthesis in *C. paspali*, we used *A. tumefaciens*-mediated transformation (Kozák et al. 2018) to separately replace these target genes in their entireties with the hygromycin resistance (*hph*) selectable marker gene. Genomic DNA of 15 hygromycin-resistant transformants each from the *idtF*- and the *idtP*-targeted mutant strains, respectively, was isolated and used as templates for a set of PCR reactions to validate the gene knockout events. Four transformants (CPIDTF2, CPIDTF3, CPIDTF7, and CPIDTF9) were found to lack the *idtF* gene, and three transformants (CPIDTP1, CPIDTP8, and CPIDTP11) were validated to miss the *idtP* gene. The wild-type *C. paspali* DSM833 and a strain transformed with the pAg-H3 plasmid were included as positive controls, and these displayed the intact *idtF* or *idtP* alleles (Fig. 3d). Further PCR reactions with primers specific for the hygromycin resistance gene and those bracketing the mutant alleles showed that the selected transformants lacking the wild-type alleles have undergone double homologous recombination replacing the *idtF* or the *idtP* gene, respectively, with the *hyg* hygromycin resistance gene (Fig. 3e and f). Genomic DNA from the wild-type strain and that transformed with the pAg-H3 vector did not yield PCR amplicons, as expected. Taken together, these PCR experiments proved that the CPIDTF2, CPIDTF3, and CPIDTF7 isolates are homokaryotic for the *ΔidtF* allele, while the CPIDTP1, CPIDTP8, and CPIDTP11 isolates are homokaryotic for the *ΔidtP* allele. These independent isolates representing the same strain (∆*idtP*, or separately ∆*idtF*) were verified to be indistinguishable during subsequent fermentations.

**Fig. 4:**
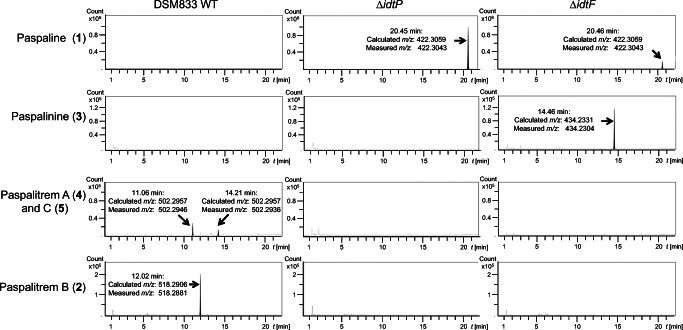
IDT profiles of the wild-type and mutant *C. paspali* strains. Extracted ion chromatograms are shown for the LC-HRMS^n^ analysis of the selected IDTs in the fermentation extracts of the wild-type *C. paspali* DSM833, the *∆idtP* mutant CPIDTP1 and the *∆idtF* mutant CPIDTF2 strains. Compounds were identified based on their exact mass with a narrow mass window (0.02 Da), and their fragmentation patterns were compared to those described in previous literature examples (Uhlig et al. 2014; Kozák et al. 2018). Peak intensities (Y axis) and chromatographic retention times (X axis) are shown with the same scale for each analyte to facilitate comparison. The representative ion chromatograms (EICs) are arranged in a matrix: the studied IDTs vary within the columns and the different strains vary within the rows (see labels on the left side of the rows and above the columns)

Next, the IDT congener profiles were determined in fermentations with the wild type, the ∆*idtP* (represented by isolates CPIDTP1 and CPIDTP8) and the ∆*idtF C. paspali* strains (represented by isolates CPIDTF2 and CPIDTF7). We searched extracts of 12 days old fermentation cultures of these strains using LC-HRMS^n^ for the presence of expected biosynthetic intermediates such as paspaline (**1**), β-PC-M6, 13-desoxypaxilline, paspalicine, paxilline, and paspalinine (**3**); and for main fermentation products such as paspalitrem A (**4**), paspalitrem C (**5**), and paspalitrem B (**2**) (Fig. 4, SI Supplementary Tables S2 and S3). As expected, the wild-type *C. paspali* DSM833 strain produced paspalitrem B (**2**, *m/z* 518.2881 for the [M+H]^+^ ion, calculated 518.2906 for C_32_H_40_NO_5_). The wild-type strain also produced two IDTs with different retention times that correspond to the structural isomers paspalitrems A and C (**4** and **5**, *m/z* 502.2946 and 502.2936 for the [M+H]^+^ ions, calculated 502.2957 for C_32_H_40_NO_4_). Intermediates of paspalitrem biosynthesis were not detected in the extracts of the wild-type strain in this set of experiments, although trace amounts of paspaline, paxilline, and paspalinine had previously been observed in similar fermentations (Kozák et al. 2018).

Analyses of fermentation extracts of the *∆idtP* strain (isolates CPIDTP1 and CPIDT1P8) revealed the absence of all paspalitrems (Fig. 4, SI Supplementary Tables S2 and S3). The only IDT congener which could be detected was paspaline (**1**). Just as with the *∆idtP* transformants, the analyses of the fermentation extracts of the *∆idtF* strain (isolates CPIDTF2 and CPIDTF9) also indicated the complete lack of prenylated IDTs. However, a new [M+H]^+^ ion at *m/z* 434.2304 was detected in the extracts (Fig. 4) at the retention time characteristic of paspalinine (**3**) and corresponding to the molecular formula of this intermediate (434.2331 for C_27_H_32_NO_4_). In addition to paspalinine (**3**), the production of small quantities of paspaline (**1**) was also observed (Fig. 4).

Besides the above mentioned biosynthetic intermediates and major products, a number of additional IDTs featuring the paspalitrem or the paspaline scaffold were also detected in *Paspalum dilatatum* samples infected with *C. paspali* (Uhlig et al. 2014). We carefully searched for the exact masses of the protonated ions of these metabolites, as well for prenylated paspaline analogues (Liu et al. 2013) in the fermentation extracts of the wild type, the *∆idtP* and the *∆idtF* strains, but none of these metabolites could be detected in the current experiments.

## Discussion

*C. paspali* produces tremorgenic paspalitrem IDT congeners in both axenic cultures and in its natural association with *Paspalum* spp. (Uhlig et al. 2014; Kozák et al. 2018). Ingestion of grass contaminated with paspalitrem mycotoxins by livestock causes serious losses for agriculture, especially in the Southern Hemisphere (Moyano et al. 2010). In our previous work, we identified the paspalitrem gene cluster of *C. paspali* by the disruption of the *idtCBGF* gene locus using *A. tumefaciens*-mediated gene disruption. As the protein products of three out of the four deleted genes are responsible for the assembly of paspaline (**1**), a common intermediate of paspaline-type IDTs, deletion of this locus resulted in the complete elimination of the full spectrum of IDTs in *C. paspali* (Kozák et al. 2018).

In the current work, we set out to substantiate bioinformatic predictions for the function of the *idtP* and *idtF* genes using specific gene knockouts that are not expected to disturb the functions of any other genes in the IDT biosynthetic gene cluster of *C. paspali*. The encoded product of the *idtP* gene is a cytochrome P450 monooxygenase with 41% sequence identity to PaxP of *P. paxilli*. In paxilline biosynthesis, PaxP mediates the oxidative conversion of paspaline (**1**, Fig. 1) to 13-desoxypaxilline. Deletion of *paxP* in *P. paxilli* resulted in the elimination paxilline production and the accumulation of the intermediate paspaline (**1**) (Saikia et al. 2007). Just as with the *∆paxP* mutation in *P. paxilli*, we found that the deletion of *idtP* in *C. paspali* leads to the accumulation of paspaline (**1**) and the complete elimination of any downstream biosynthetic products such as paspalinine (**3**) and paspalitrems A, B, and C (**2**, **4**, and **5**). Importantly, the inactivation of *idtP* orthologues in other IDT producer fungi yielded different results. Thus, disruption of *janP* in the shearinine producer *P. janthinellum* resulted in the elimination of the production of the full spectrum of IDTs, including the production of paspaline (**1**) that is the substrate of the JanP cytochrome P450 monooxygenase (Nicholson et al. 2015). This indicates that the overall regulation of the assembly of the IDT scaffold differs in *P. janthinellum* from that of *C. paspali* and other IDT producers such as *P. paxilli*. Even more surprisingly, inactivation of the *idtP* orthologue *terP* in the terpendole producer *T. album* leads to the accumulation of terpendole E, a C11-hydroxylated derivative of paspaline (**1**), and the appearance of a new shunt metabolite, 11-ketopaspaline. This indicates that during the biosynthesis of the terpendoles (and by extension, that of the lolitrems), oxidation of paspaline (**1**) catalyzed by TerQ (and LtmQ for lolitrems) precedes that of TerP (and LtmP), in contrast to the biosynthetic order of the orthologous enzymes in the rest of the paspaline-derived IDTs such as paspalitrems, aflatrems, shearinines, penitrems, sulpinines, and janthitrems (Saikia et al. 2012; Motoyama et al. 2012).

Among the various IDTs, paspalitrems are most similar to and constitute the structural isomers of the aflatrems that are produced by *Aspergillus flavus* (Cole et al. 1977; Gallagher and Wilson 1979). Thus, normal prenylation of the indole moiety of paspalinine (**3**) at the C21 and C20 positions yields the 2-methylbut-2-ene side chain in paspalitrems A and C, respectively (**4** and **5**, Fig. 1). For aflatrems, reverse prenylation of paspalinine at the same positions affords the 3-methylbut-1-ene side chains of β-aflatrem and aflatrem A, respectively. IdtF of *C. paspali* is a predicted prenyltransferase with 21% identity over 78% of the amino acid sequence to AtmP encoded in the aflatrem gene cluster of *A. flavus* that is responsible for the reverse prenylation of paspalinine (**3**) (Liu et al. 2013). IdtF shows similar levels of identities to PtmD and PenD (both 21% over 95% coverage at the amino acid level), normal prenyltransferases that modify β-paxitriol during penitrem production in *Penicillium* strains (Oikawa et al. 2016). We proposed that IdtF in *C. paspali* is responsible for the normal prenylation of paspalinine (**3**) at the C21 or C20 positions to afford paspalitrem A or C (**4** or **5**), respectively (Kozák et al. 2018). In agreement with this, the *∆idtF* knockout strain of *C*. *paspali* was unable to produce prenyl-elaborated IDTs, and accumulated paspalinine (**3**) with minor amounts of paspaline (**1**) also detectable. This result clearly validates the predicted function of IdtF as a paspaline C20 or C21 normal prenyltransferase. This also indicates that normal prenylation of paspalinine (**3**) (as with IdtF) vs. reverse prenylation of the same substrate (as with AtmD) demanded the parallel evolution of non-orthologous prenyltransferases despite the identical substrate. On the other hand, normal prenyltransferases such as IdtF vs. PtmD or PenD are also highly divergent, reflecting the different substrates (paspalinine **3** vs. β-paxitriol).

Purified AtmD from *A. flavus* and PaxD from *P. paxilli* were reported to accept paspaline (**1**) as an alternative substrate in vitro and catalyze the formation of paspaline derivatives *α*-prenylated at the C21 or C22 positions (Fig. 1) (Liu et al. 2013). Since the *C. paspali ∆idtP* strain accumulates paspaline (**1**) and has an intact copy of the *idtF* gene, we considered that the prenyltransferase IdtF may also be able to produce prenylated paspaline derivatives. We also considered the possibility that the prenyltransferase DmaW from the ergot alkaloid biosynthetic gene cluster of *C. paspali* (27.4% identity over 92% coverage at the amino acid level to AtmD) may engage in metabolic crosstalk to produce prenylated paspaline derivatives. However, no metabolites could be detected with the calculated m/z of monoprenyl-paspaline or hydroxyprenyl-paspaline in the fermentation extracts of the wild type or the *∆idtP* strains. This result indicates that at least in vivo, neither IdtF nor DmaW would accept paspaline (**1**) as a substrate, although just as with AtmD and PaxD, purified IdtF or DmaW may catalyze paspaline monoprenylation with a low efficiency in vitro.

Uhlig and coworkers (Uhlig et al. 2014) showed that in addition to the expected biosynthetic intermediates of paspalitrem biosynthesis, field samples of *C. paspali* (such as the *C. paspali*-*P. dilatatum* association) also produce a number of compounds with the paspaline or paspalitrem scaffold*.* Thus, we investigated the presence of such analogues in axenic cultures of *C. paspali* DSM833 and its *∆idtP* and *∆idtF* knockout strains. However, none of these analogues (SI Supplementary Table S2) was detectable. This discrepancy between the IDT profiles of the field samples vs. the axenic cultures may be explained by dissimilar regulation of gene expression and/or the presence of plant-derived enzymes and the extended cultivation period in the case of field-collected samples.

In this study, we demonstrated that targeted disruption of the *idtP* and *idtF* genes in *C. paspali* modulates the IDT product spectrum towards paspaline-type IDTs such as paspaline (**1**) and paspalinine (**3**). These results highlight the metabolic engineering potential of the *A. tumefaciens*-mediated *C. paspali* transformation system (Kozák et al. 2018) in creating efficient platforms for the production of the IDT nucleus for combinatorial biosynthesis and the large-scale production of complex IDTs for various biomedical applications in the future (Kozák et al. 2019).

## Electronic supplementary material

ESM 1 (DOCX 261 kb)

## References

[CR1] Arcamone F, Bonino C, Chain EB, Ferretti A, Pennella P, Tonolo A, Vero L (1960). Production of lysergic acid derivatives by a strain of *Claviceps paspali* Stevens and Hall in submerged culture. Nature.

[CR2] Cawdell-Smith A, Scrivener C, Bryden W (2010). Staggers in horses grazing paspalum infected with *Claviceps paspali*. Aust Vet J.

[CR3] Cole RJ, Dorner JW, Lansden JA, Cox RH, Pape C, Cunfer B, Nicholson SS, Bedell DM (1977). Paspalum staggers: isolation and identification of tremorgenic metabolites from sclerotia of *Claviceps paspali*. J Agric Food Chem.

[CR4] Gallagher RT, Wilson BJ (1979). Aflatrem, the tremorgenic mycotoxin from *Aspergillus flavus*. Mycopathologia.

[CR5] Gritz L, Davies J (1983). Plasmid-encoded hygromycin B resistance: the sequence of hygromycin B phosphotransferase gene and its expression in *Escherichia coli* and *Saccharomyces cerevisiae*. Gene.

[CR6] Kozák L, Szilágyi Z, Vágó B, Kakuk A, Tóth L, Molnár I, Pócsi I (2018). Inactivation of the indole-diterpene biosynthetic gene cluster of *Claviceps paspali* by *Agrobacterium*-mediated gene replacement. Appl Microbiol Biotechnol.

[CR7] Kozák L, Szilágyi Z, Tóth L, Pócsi I, Molnár I (2019). Tremorgenic and neurotoxic paspaline-derived indole-diterpenes: biosynthetic diversity, threats and applications. Appl Microbiol Biotechnol.

[CR8] Liu C, Minami A, Noike M, Toshima H, Oikawa H, Dairi T (2013). Regiospecificities and prenylation mode specificities of the fungal indole diterpene prenyltransferases AtmD and PaxD. Appl Environ Microbiol.

[CR9] Liu C, Tagami K, Minami A, Matsumoto T, Frisvad JC, Suzuki H, Ishikawa J, Gomi K, Oikawa H (2015). Reconstitution of biosynthetic machinery for the synthesis of the highly elaborated indole diterpene penitrem. Angew Chem Int Ed.

[CR10] McMillan LK, Carr RL, Young CA, Astin JW, Lowe RGT, Parker EJ, Jameson GB, Finch SC, Miles CO, McManus OB, Schmalhofer WA, Garcia ML, Kaczorowski GJ, Goetz M, Tkacz JS, Scott B (2003). Molecular analysis of two cytochrome P450 monooxygenase genes required for paxilline biosynthesis in *Penicillium paxilli*, and effects of paxilline intermediates on mammalian maxi-K ion channels. Mol Gen Genomics.

[CR11] Motoyama T, Hayashi T, Hirota H, Ueki M, Osada H (2012). Terpendole E, a kinesin Eg5 inhibitor, is a key biosynthetic intermediate of indole-diterpenes in the producing fungus *Chaunopycnis alba*. Chem Biol.

[CR12] Moyano M, Molina A, Lora A, Mendez J, Rueda A (2010). Tremorgenic mycotoxicosis caused by *Paspalum paspaloides* (Michx.) Scribner infected by *Claviceps paspali*: a case report. Veterinární Medicína.

[CR13] Nicholson MJ, Koulman A, Monahan BJ, Pritchard BL, Payne GA, Scott B (2009). Identification of two aflatrem biosynthesis gene loci in *Aspergillus flavus* and metabolic engineering of *Penicillium paxilli* to elucidate their function. Appl Environ Microbiol.

[CR14] Nicholson M, Eaton C, Stärkel C, Tapper B, Cox M, Scott B (2015). Molecular cloning and functional analysis of gene clusters for the biosynthesis of indole-diterpenes in *Penicillium crustosum* and *P. janthinellum*. Toxins.

[CR15] Oikawa H, Minami A, Liu C (2016). Total biosynthesis of fungal indole diterpenes using cell factories. Heterocycles.

[CR16] Saikia S, Parker EJ, Koulman A, Scott B (2007). Defining paxilline biosynthesis in *Penicillium paxilli*: functional characterization of two cytochrome P450 monooxygenases. J Biol Chem.

[CR17] Saikia S, Takemoto D, Tapper BA, Lane GA, Fraser K, Scott B (2012). Functional analysis of an indole-diterpene gene cluster for lolitrem B biosynthesis in the grass endosymbiont *Epichloë festucae*. FEBS Lett.

[CR18] Tarui Y, Chinen T, Nagumo Y, Motoyama T, Hayashi T, Hirota H, Muroi M, Ishii Y, Kondo H, Osada H, Usui T (2014). Terpendole E and its derivative inhibit STLC- and GSK-1-resistant Eg5. ChemBioChem.

[CR19] Tudzynski P, Correia T, Keller U (2001). Biotechnology and genetics of ergot alkaloids. Appl Microbiol Biotechnol.

[CR20] Uhlig S, Egge-Jacobsen W, Vrålstad T, Miles CO (2014). Indole-diterpenoid profiles of *Claviceps paspali* and *Claviceps purpurea* from high-resolution Fourier transform Orbitrap mass spectrometry. Rapid Commun Mass Spectrom.

[CR21] Young CA, Bryant MK, Christensen MJ, Tapper BA, Bryan GT, Scott B (2005). Molecular cloning and genetic analysis of a symbiosis-expressed gene cluster for lolitrem biosynthesis from a mutualistic endophyte of perennial ryegrass. Mol Gen Genomics.

[CR22] Young CA, Felitti S, Shields K, Spangenberg G, Johnson RD, Bryan GT, Saikia S, Scott B (2006). A complex gene cluster for indole-diterpene biosynthesis in the grass endophyte *Neotyphodium lolii*. Fungal Genet Biol.

[CR23] Zhang A, Lu P, Dahl-Roshak A, Paress P, Kennedy S, Tkacz J, An Z (2003). Efficient disruption of a polyketide synthase gene ( pks1) required for melanin synthesis through *Agrobacterium*-mediated transformation of *Glarea lozoyensis*. Mol Gen Genomics.

