# Supplementary material for: Functional characterization of the idtF and idtP genes in the Claviceps paspali indole diterpene biosynthetic gene cluster
Source: Folia Microbiol (Praha). 2020 Feb 19;65(3):605–13. doi: 10.1007/s12223-020-00777-6 (PMC7244603; doi:10.1007/s12223-020-00777-6)
Supplement: Supplementary file 1 — (DOCX 261 kb) [file 12223_2020_777_MOESM1_ESM.docx]

# SUPPLEMENTARY INFORMATION FOR

# Functional characterization of the *idtF* and *idtP* genes in the *Claviceps paspali* indole diterpene biosynthenthetic gene cluster

Short Communication

László Kozák^1,2^, Zoltán Szilágyi^2^, László Tóth^2^, István Pócsi^1,*^, and István Molnár^3^

^1^Department of Molecular Biotechnology and Microbiology, Institute of Biotechnology, Faculty of Science and Technology, University of Debrecen, Debrecen, Hungary

^2^Teva Pharmaceutical Works Ltd., Debrecen, Hungary

^3^Southwest Center for Natural Products Research, School of Natural Resources and the Environment, University of Arizona, Tucson, USA

Corresponding author:

*I. Pócsi, e-mail: pocsi.istvan@science.unideb.hu, telephone: +36-52-512900 ext. 62337, ORCID ID: 0000-0003-2692-6453

**Supplementary Table S1. Primers used in this study.**

| Name | Primer sequence^1^ | Primer size (bp) | DNA strand direction |
| --- | --- | --- | --- |
| pAg-F | 5'-gtcgcataagggagagcgtc-3' | 20 | Forward |
| pAg-R | 5'-tgcgcagcctgaatggcgaa-3' | 20 | Reverse |
| *hph*-F | 5'-gtcgacagaagatgatattgaaggagcatt-3' | 30 | Forward |
| *hph*-R | 5'-gaggatcctctagaaagaaggattacctct-3' | 30 | Reverse |
| *idtF*-LTA-F | 5'-cgccattcgccattcaggctgcgcacatacattgattagtctaaagaggatggtc-3' | 55 | Forward |
| *idtF*-LTA-R | 5'-tccttcaatatcatcttctgtcgacctttactgccactctacgagcaactcattc-3' | 55 | Reverse |
| *idtF*-RTA-F | 5'-atccttctttctagaggatcctctacagcattggctttcgaggtcgtgttgat-3' | 55 | Forward |
| *idtF*-RTA-R | 5'-ccgtcgacgctctcccttatgcgacctttctgtcttgacacatccaacaaaacag-3' | 55 | Reverse |
| *idtP*-LTA-F | 5'-cgccattcgccattcaggctgcgcagtggggacaggtggcgtggctatcagggcc-3' | 55 | Forward |
| *idtP*-LTA-R | 5'-tccttcaatatcatcttctgtcgacgcgtttcctcgcttcggtacgttgctttca-3' | 55 | Reverse |
| *idtP*-RTA-F | 5'-atccttctttctagaggatcctcattccgacggtgcgttgcgcgagggcagtt-3' | 55 | Forward |
| *idtP*-RTA-R | 5'-ccgtcgacgctctcccttatgcgaccatcccgtcaaacagataagacaggtgcat-3' | 55 | Reverse |
| *hph*-in-F | 5'-cgtatatgctccgcattggt-3' | 20 | Forward |
| *hph*-in-R | 5'-aagcacttccggaatcggg-3' | 19 | Reverse |
| *idtF*-out-F | 5'-agaaaaactctttccgatcggctc-3' | 24 | Forward |
| *idtF*-out-R | 5'-atagacggagacgcagattgcgta-3' | 24 | Reverse |
| *idtP*-out-F | 5'-ccgcagagacgatccagcta-3' | 20 | Forward |
| *idtP*-out-R | 5'-tttggcatgtcgatatctctttctg-3' | 25 | Reverse |

^1^Underlined bases introduce an overlap with the next PCR amplicon for Gibson assembly.

**Supplementary Table S2. IDTs analyzed in this study.**

| **Compound name** | **Structure** | **Calculated *m/z* for the [M+H]^+^ ion** | **Elemental composition for the [M+H]^+^ ion** | **Reference** |
| --- | --- | --- | --- | --- |
| **Intermediates of paspalitrem biosynthesis** | | | | |
| Emindole SB |  | 406.3109 | C_28_H_40_NO | (Nozawa et al. 1988) |
| Paspaline (**1**) |  | 422.3059 | C_28_H_40_NO_2_ | (Fehr and Acklin 1966) |

| PC-M6 |  | 422.2695 | C_27_H_36_NO_3_ | (Hosoe et al. 1990) |
| --- | --- | --- | --- | --- |
| 13-desoxypaxilline |  | 420.2538 | C_27_H_34_NO_3_ | (Nozawa et al. 1988) |
| Paxilline |  | 437.2566 | C_27_H_35_NO_4_ | (Cole et al. 1974) |

| Paspalicine |  | 418.2382 | C_27_H_32_NO_3_ | (Fehr and Acklin 1966) |
| --- | --- | --- | --- | --- |
| Paspalinine (**3**) |  | 434.2331 | C_27_H_32_NO_4_ | (Fehr and Acklin 1966) |
| Paspalitrem A (**4**) |  | 502.2957 | C_32_H_40_NO_4_ | (Cole et al. 1977) |

| Paspalitrem C (**5**) |  | 502.2957 | C_32_H_40_NO_4_ | (Dorner et al. 1984) |
| --- | --- | --- | --- | --- |
| Paspalitrem B (**2**) |  | 518.2906 | C_32_H_40_NO_5_ | (Cole et al. 1977) |

| **Metabolites with a paspalitrem or a paspaline scaffold isolated from the *Paspalum spp. – C. paspali* association** | | | | |
| --- | --- | --- | --- | --- |
| Putative paspalitrem analogue^1^ |  | 502.2957 | C_32_H_40_NO_4_ | (Uhlig et al. 2014) |
| Putative paspaline analogue |  | 452.28 | C_28_H_38_NO_4_ | (Uhlig et al. 2014) |
| Putative paspalitrem analogue |  | 506.327 | C_32_H_44_NO_4_ | (Uhlig et al. 2014) |

| Putative paspaline analogue |  | 422.2695 | C_27_H_36_NO_3_ | (Uhlig et al. 2014) |
| --- | --- | --- | --- | --- |
| Putative paspaline analogue |  | 438.3008 | C_28_H_40_NO_3_ | (Uhlig et al. 2014) |
| Putative paspaline analogue |  | 420.2538 | C_27_H_34_NO_3_ | (Uhlig et al. 2014) |

| Putative paspalitrem analogue |  | 500.28 | C_32_H_38_NO_4_ | (Uhlig et al. 2014) |
| --- | --- | --- | --- | --- |
| Putative paspalitrem analogue |  | 490.3321 | C_32_H_44_NO_3_ | (Uhlig et al. 2014) |
| Putative paspalitrem analogue |  | 486.3008 | C_32_H_40_NO_3_ | (Uhlig et al. 2014) |

| **Paspaline analogues prenylated by AtmD or PaxD** | | | | |
| --- | --- | --- | --- | --- |
| C21-prenylpaspaline |  | 490.3685 | C_33_H_48_NO_2_ | (Liu et al. 2013) |
| C22-prenylpaspaline |  | 490.3685 | C_33_H_48_NO_2_ | (Liu et al. 2013) |
| Hydroxyprenyl-paspaline |  | 506.3634 | C_33_H_48_NO_3_ | Hypothetical compound |

^1^The elemental composition (and thus the calculated *m/z*) of the protonated ion of this compound is identical to that of paspalitrem A (**4**) and C (**5**).

**Supplementary Table S3. Main IDT metabolites in fermentation extracts of *C. paspali* strains.**

| **Compound name** | **Wild type DSM833** | ***ΔidtP* mutant** | ***ΔidtF* mutant** |
| --- | --- | --- | --- |
| Paspaline, **1** | - | + | + |
| Paspalinine, **3** | - | - | + |
| Paspalitrem A and C, **4** and **5** | + | - | - |
| Paspalitrem B, **2** | + | - | - |
